# Supplementary material for: Thirty-day hospital readmission in females with acute heart failure and breast cancer: A retrospective cohort study from national readmission database
Source: PLoS One. 2024 Jul 23;19(7):e0301596. doi: 10.1371/journal.pone.0301596 (PMC11265691; doi:10.1371/journal.pone.0301596)
Supplement: S1 Table — (DOCX) [file pone.0301596.s001.docx]

| **CONDITION** | **ICD CODE** |
| --- | --- |
| Acute Heart Failure | I50.21, I50.23, I50.31, I50.33, I50.41, I50.43 |
| Hypertension | I10 |
| Hypertensive Chronic Kidney disease | I12.9, I12.0 |
| Hypertensive heart and chronic kidney disease with heart failure, with stage 1 through stage 4 chronic kidney disease, or unspecified chronic kidney disease | I13.0 |
| Hypertensive heart and chronic kidney disease without heart failure | I13.1 |
| Hypertensive heart and chronic kidney disease without heart failure, with stage 5 chronic kidney disease, or end stage renal disease | I13.11, I13.2 |
| Renovascular hypertension | I15.0 |
| Other secondary hypertension | I15.8, I6.74 |
| Hypertensive heart and chronic kidney disease without heart failure | I13.1 |
| Hypertensive heart and chronic kidney disease without heart failure, with stage 5 chronic kidney disease, or end stage renal disease | I13.11, I13.2 |
| Renovascular hypertension | I15.0 |
| Other secondary hypertension | I15.8, I6.74 |

Breast cancer ICD codes-

C50.011, C50.012, C50.019, C50021, C50022, C50029, C50111, C50112, C50119, C50121, C50122, C50129, C50211, C50212, C50219, C50221, C50222, C50229, C50311, C50312, C50319, C50321, C50322, C50329, C5041, C50412, C50419, C50421, C50422, C50429, C50511, C50512, C50519, C50521, C50522, C50529, C50611, C50612, C50619, C50621, C50622, C50629, C50811, C50812, C50819, C50821, C50822, C50829, C50911, C50912, C50919, C50921, C50922, C50929
