# Supplementary material for: Thirty-day hospital readmission in females with acute heart failure and breast cancer: A retrospective cohort study from national readmission database
Source: PLoS One. 2024 Jul 23;19(7):e0301596. doi: 10.1371/journal.pone.0301596 (PMC11265691; doi:10.1371/journal.pone.0301596)
Supplement: Attachment — Submitted filename: Response to Reviewers.docx [file pone.0301596.s002.docx]

**Dear Editor-in-Chief and Reviewers,**

We submit for the revision of our manuscript entitled " **Thirty-day Hospital Readmission in Females with Acute Heart Failure and Breast Cancer: A Retrospective Cohort Study from National Readmission Database”** for consideration in your respected journal.

We are grateful for your critical comments and thoughtful suggestions which are valuable and very helpful for revising and improving our paper. We truly appreciate your time, effort, and patience towards providing these queries and suggestions.

The following is a point-by-point response to the reviewers’ comments.

**Reviewer #1:**

**Question:  Introduction: Would add more info on prevalence of HF in Breast cancer patients? what cardiovascular causes are the major cause of death in Breast cancer patients- like CHF/MI etc. This would explain why tis study is important
Almost-one-in =four (this is hw it appeared on manuscript for review). please check?**

Response: Thank you for the query and suggestions.

We have reviewed literature on HF prevalence in Breast cancer and added it to the introduction as suggested. Also made appropriate changes regarding the incidence of cardiovascular complications in breast cancer patients.

**Reviewer Question:**

**Study design:
- If AHF was the principal diagnosis - does it mean just ICD codes for acute heart failure(I50)? If thats the case then what about hypertensive heart and chronic kidney disease with heart failure, code I13. This diagnosis is more often the principal in pts with HF and ckd. Was the ICD code for this used?
- Need to update what icd10 codes were used (for heart failure)
- So were patients with history of breast cancer who were in remission excluded for this study purpose?**

Response: Thank you for the query and suggestions.

Following ICD codes were used for Acute heart failure, Hypertensive heart disease and chronic kidney disease for our study’s analysis.

Below is the table depicting the ICD codes used for analysis.

| CONDITION | ICD CODE |
| --- | --- |
| Acute Heart Failure | I50.21, I50.23, I50.31, I50.33, I50.41, I50.43 |
| Hypertension | I10 |
| Hypertensive Chronic Kidney disease | I12.9, I12.0 |
| Hypertensive heart and chronic kidney disease with heart failure, with stage 1 through stage 4 chronic kidney disease, or unspecified chronic kidney disease | I13.0 |
| Hypertensive heart and chronic kidney disease without heart failure | I13.1 |
| Hypertensive heart and chronic kidney disease without heart failure, with stage 5 chronic kidney disease, or end stage renal disease | I13.11, I13.2 |
| Renovascular hypertension | I15.0 |
| Other secondary hypertension | I15.8, I6.74 |
| Hypertensive heart and chronic kidney disease without heart failure | I13.1 |
| Hypertensive heart and chronic kidney disease without heart failure, with stage 5 chronic kidney disease, or end stage renal disease | I13.11, I13.2 |
| Renovascular hypertension | I15.0 |
| Other secondary hypertension | I15.8, I6.74 |

**Reviewer Question: So, were patients with history of breast cancer who were in remission excluded for this study purpose?**

Response: Thank you for the query.

All patients with history or active breast cancer were included in the study with following ICD codes.

Breast cancer ICD codes-

C50.011, C50.012, C50.019, C50021, C50022, C50029, C50111, C50112, C50119, C50121, C50122, C50129, C50211, C50212, C50219, C50221, C50222, C50229, C50311, C50312, C50319, C50321, C50322, C50329, C5041, C50412, C50419, C50421, C50422, C50429, C50511, C50512, C50519, C50521, C50522, C50529, C50611, C50612, C50619, C50621, C50622, C50629, C50811, C50812, C50819, C50821, C50822, C50829, C50911, C50912, C50919, C50921, C50922, C50929

**Reviewer Question: hypertensive heart disease with chronic kidney disease seems to be the major cause of readmission. As mentioned about hypertensive heart disease with chronic kidney disease with heart failure(I30) is the principal diagnosis in most patients who have CKD as well so it's important to know if this ICD10 was used for extracting data in first place?**

Response: Thank you for the query and suggestions.

Yes, we have included both hypertensive heart disease and chronic kidney diseases ICD codes for study’s data extraction.

Hypertensive Chronic Kidney disease- I12.9, I12.0

Hypertensive heart and chronic kidney disease with heart failure (with stage 1 through stage 4 chronic kidney disease, or unspecified chronic kidney disease)-I13.0

### Hypertensive heart and chronic kidney disease without heart failure- I13.1

Hypertensive heart and chronic kidney disease without heart failure (with stage 5 chronic kidney disease, or end stage renal disease)- I13.11, I13.2

**Reviewer Question:**

**Discussion
- first para seems repeat of what was already said above in results
- second para needs to be modified - seems widely jumping from one to other- initially talk about Several studies have investigated a robust correlation between the likelihood of developing HF and breast cancer". Then at end of the para states "underlying association between hypertension and the risk of breast cancer has not been extensively researched or studied yet". This study if apt CHF and breast cancer so narrow down to that- pathophysiology, what evidence other studies show. Also talks about medicare pts, REGARD study which is more about stroke rather than HF. Please use data about Medicare patients and HF readmission if needed.**

Response: Thank you for the suggestions.

We have revised the first paragraph of the discussion as advised. We have also reviewed existing literature and made changes in the second paragraph to address their findings correlating with our analysis. We included the findings of REGARDS study as it reported the impact of socioeconomic parameters, social determinants of life (poverty, insurance type, education, household income) in medicare beneficiaries on the long-term outcomes, morbidity and rehospitalization rates in HF patients.

REGARDS STUDY by Sterling, et.al

Sterling MR, Ringel JB, Pinheiro LC, et al. Social Determinants of Health and 30-Day Readmissions Among Adults Hospitalized for Heart Failure in the REGARDS Study. *Circ Heart Fail*. 2022;15(1):e008409.

**Reviewer #2: increase readmission rate as expected on the lines of other research studies/ literature we already have
if you can try to add other variables if available would be more novel.
overall good writing and research methodology used.**

Response: Thank you for your kind words and suggestions.

We aimed at studying readmission rates for heart failure patients in breast cancer using National Readmission Database. Our objective, as a nascent research team, was to investigate the impact of medical comorbidities and socioeconomic parameters in relation to HF readmission rates in breast cancer. Your input is invaluable, and we surely plan to incorporate additional variables into our future studies based on your recommendations.
